# Paracentesis in Empyema

**Published:** 1890-03-08

**Authors:** 


### PARACENTESIS IN EMPYEMA.

Küster enters a vigorous protest against the conclusions of the Wiesbaden Congress, that paracentesis is contra-indicated in cases of a tubercular or phthisical character, that in others several consecutive tappings may be performed, that resection of a rib should be restricted to those in which the pus is sanious, and that the point selected should be in the 4th or 5th intercostal space in the axillary line.

He, on the contrary, would perform the radical operation in nearly all empyemas, even tubercular, resecting the rib in the lowest point posteriorly, and in every case, even where there is a bronchial fistula, with the fullest antiseptic precautions.

For ten years his practice has been to make an incision anteriorly in the 4th or 5th intercostal space, close to the upper border of the rib, so as to avoid wounding the artery. While the pus is flowing out he passes in a silver sound, which he pushes backwards until he finds the most dependent point, where he excises a piece of the rib that a free exit may be secured. After irrigation under gentle pressure, and clearing the orifices of coagula by means of a pointed sponge he passes a drainage tube from before backwards. Of 109 cases so treated 60 were cured, 17 remained uncured, and 32 died. These figures are not so unfavourable as they might at first sight appear, for no cases were rejected as unsuitable. Thirty-nine were of advanced tubercular phthisis, and nine of these were cured so far as the empyema was concerned. Of the 32 deaths only six or seven could be in any way attributed to the operation, 25 dying of pre-existing disease of the lung.

The operation is not particularly dangerous. The risks to be avoided are hæmorrhage which can nearly always be easily arrested, and, at least in cases of great exudation, narcosis, in which he advises cautious and very moderate use of chloroform, and only in the earlier part of the operation. Also such a posture that the exudation may not weigh on the sound side and the heart. The anterior opening he makes while the patient is lying comfortably on his back, the posterior in a half-turned or sitting posture.

